# It's just evolution. Commentary: A crisis in comparative psychology: where have all the undergraduates gone?

**DOI:** 10.3389/fpsyg.2015.01873

**Published:** 2015-12-02

**Authors:** Madeleine I. R. Brodbeck, David R. Brodbeck

**Affiliations:** ^1^Department of Psychology, The University of Western OntarioLondon, ON, Canada; ^2^Department of Psychology, Algoma UniversitySault Ste. Marie, ON, Canada

**Keywords:** comparative psychology, evolution, undergraduate students, cognition, recruitment, comparative cognition

Abramson (2015) presents a series of arguments that support the notion that there is a crisis in comparative psychology. We have chosen to concentrate on one of his arguments, specifically the idea that comparative psychology is in crisis because nobody calls it comparative psychology anymore. Indeed, it seems that Abramson (2015) is concerned mostly with the name of the discipline, and not with the questions being asked.

## From hodos and campbell to shettleworth

In 1969 Hodos and Campbell asked the question “Why is there no theory in comparative psychology?” This was echoed by Sara Shettleworth (1993) who asked the question “Where is the comparison in comparative cognition?” Both of these papers, and a number of others (e.g., Kamil, 1987) pointed out that just doing comparisons for the sake of comparisons (e.g., do rats or pigeons show the same sort of patterns in their behavior, or do they show the same sort of effects we see in humans) indicate an implicit question. That question is “are rats or pigeons like people?”

Hodos and Campbell (1969) pointed out that such questions show a fundamental misunderstanding of how evolution works. That fundamental misunderstanding is the idea of a *scala naturae*, a rank ordering of species. Kamil (1987) pointed out that it is much more reasonable, and fruitful if we look at natural history as a starting point for our comparative questions. For example, one might expect a difference between food-storing and non-storing birds in their use of spatial memory, at the expense of other types of memory, to solve problems (e.g., Brodbeck, 1994) but one should not expect differences between storers and non-storers on their use of memory for color to solve a problem.

This approach, called “the synthetic approach” by Kamil (1987) or “the ecological approach” by Shettleworth (1993) has gained favor over the past few decades over what Shettleworth termed “the anthropocentric approach.”

If one accepts that this newer, evolutionarily driven approach has become the norm in comparative psychology it becomes clear why we see comparative questions attacked from multiple directions, and from researchers with many different kinds of training. So, the idea that “comparative cognition,” “evolutionary psychology,” “integrative biology,” or “behavioral ecology” has sort of subsumed traditional “comparative psychology” is not really surprising. This is part of the maturation of the field, and the maturation of the types of answers researchers seek to comparative questions. This is exactly what is predicted if one takes the ideas of Hodos and Campbell (1969), Kamil (1987), and Shettleworth (1993) as sensible.

We see then, no real problem, or crisis, here. Instead what we see is a sub field of experimental psychology growing to include approaches from many different types of researchers. We see here something that occurs naturally, as a science progresses. Comparative psychology, comparative cognition, or whatever one wishes to call it, has become a fundamental approach to the evolution of the scientific analyses of behavior and cognition.

## Funding

This research was funded by a Natural Science and Engineering Research Council of Canada Graduate Scholarship to MB and by the Algoma University Travel and Research Fund to DB.

### Conflict of interest statement

The authors declare that the research was conducted in the absence of any commercial or financial relationships that could be construed as a potential conflict of interest.
